# Dietary effect of low fish meal aquafeed on gut microbiota in olive flounder (*Paralichthys olivaceus*) at different growth stages

**DOI:** 10.1002/mbo3.992

**Published:** 2020-01-11

**Authors:** Kai‐Min Niu, Bong‐Joo Lee, Damini Kothari, Woo‐Do Lee, Sang‐Woo Hur, Sang‐Gu Lim, Kang‐Woong Kim, Kyoung‐Duck Kim, Na‐Na Kim, Soo‐Ki Kim

**Affiliations:** ^1^ Institute of Biological Resources Jiangxi Academy of Sciences Nanchang China; ^2^ Department of Animal Science and Technology Konkuk University Seoul Korea; ^3^ Aquafeed Research Center National Institute of Fisheries Science Pohang Korea; ^4^ Aquaculture Management Division National Institute of Fisheries Science Busan Korea; ^5^ Inland Aquaculture Research Center National Institute of Fisheries Science Changwon Korea

**Keywords:** growth stage, gut microbiota, low fish meal, next‐generation sequencing, olive flounder

## Abstract

This study was conducted to investigate the long‐term effect of a low fish meal (FM) diet comprising plant‐based protein sources (PPS) on changes of gut microbial diversity in olive flounder (*Paralichthys olivaceus*) over the course of life. Two experimental diets were prepared to contain 74% FM (control) or 52% FM with 22% PPS (30% FM replacement, FM30). Fish were fed one of the two experimental diets for 8 months, and we collected the midgut contents to analyze the gut bacterial community by Illumina MiSeq based on the metagenomic sequences in the V3–V4 regions of 16S rRNA. We found that there were nine dominant phyla, which in turn presented Proteobacteria, Firmicutes, and Actinobacteria as the three major phyla in the gut microbiota of the flounder. At genus level, the dominant genera were *Delftia*, *Prevotella*, and *Chthoniobacter* at the juvenile stage (below 100 g/fish); *Chthoniobacter*, *Bacillus*, and *Bradyrhizobium* at the grower stage (400 g/fish); *Chthoniobacter*, *Bacillus*, and *Delftia* at the subadult stage (800 g/fish); and *Lactobacillus* and *Prevotella* at the adult stage (over 1,000 g/fish). The microbial diversity in olive flounders arched from the juvenile and subadult stage and reached a plateau thereafter. The fish fed the FM30 diet significantly had an increased abundance of *Lactobacillus* and *Photobacterium* and had less abundance of *Prevotella* and *Paraprevotella* than the control. However, the effect of dietary PPS was not significant on total microbial richness, indicating no negative effect as feed sources on the intestinal microbiota in olive flounder. These results indicate that the life stage of olive flounder is more important in modulating intestinal microbiota than is the diet. It could also be concluded that dietary PPS might be used as a potential fish meal alternative without any compromising effects on microbial diversity of olive flounder for long‐term feeding.

## Graphical abstract

This study was conducted to investigate the long‐term effect of a low fish meal diet FM30 comprising plant‐based protein sources (PPS) on changes of gut microbial diversity in olive flounder (*Paralichthys olivaceus*) over the course of life. The data indicate that the life stage is more important in modulating intestinal microbiota than is the diet. The dietary PPS might be used as a potential fish meal alternative without any compromising effects on microbial diversity of olive flounder for long‐term feeding.

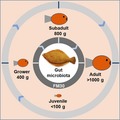

## 1 INTRODUCTION

Olive flounder (*Paralichthys olivaceus*) is the most economically important carnivorous marine fish cultured in Republic of Korea, which has achieved more than 45% of all fish production (KOSTAT, 2017). Fish meal (FM) remains the gold‐standard protein ingredient for carnivorous aquaculture fish. The high proportion of FM is generally used to support the growth and health in the juvenile stage, and then, it is progressively reduced during the grow‐out stage (~30–40 weeks; Naylor et al., 2009). The lower supply together with the huge demand has resulted in a sharp rise in FM price globally. To reduce the reliance on using FM in aquafeed and keep sustainable development of Korean aquaculture in the future, researchers and producers have attempted to establish cost‐effective FM alternatives. Plant protein origins have advantages in price, stable supply, and nutritional composition (Daniel, 2018; Gatlin et al., 2007). A wide range of plant protein ingredients such as soybean (Murashita et al., 2018; Ye et al., 2019; Zhang et al., 2018), corn gluten (Gerile & Pirhonen, 2017), corn‐protein concentrate (Ng, Leow, & Yossa, 2019), wheat gluten (Monge‐Ortiz et al., 2016), rapeseed (Dossou et al., 2018), peas (Nogales‐Mérida, Tomás‐Vidal, Moñino‐López, Jover‐Cerdá, & Martínez‐Llorens, 2016), canola (Thiessen, Maenz, Newkirk, Classen, & Drew, 2004), cottonseed (Pham et al., 2008; Pham, Lee, Lim, & Park, 2007), and rice distillers' dried grain (Bae, Kim, & Lee, 2015) have been explored as FM alternatives in aquafeed. However, the presence of antinutritional factors or nutritional imbalance in the plant proteins that might negatively affect fish growth, gut microbiota composition, immune response, and survivability (Desai et al., 2012; Liang et al., 2019). Recently, replacement of 30% FM with soybean meal (SBM) has been reported as not influencing the growth gain and specific growth rate of obscure puffer within 8 weeks of feeding (Ye et al., 2019). The potential of fermented soybean and corn gluten as FM alternatives has been evaluated in olive flounder (Seong et al., 2018). The inclusion of <40% of these plant proteins showed no negative effect on the growth, hematology, and nonspecific immune response in olive flounder over a period of 8 weeks. Making a high‐level replacement of FM or free‐FM aquafeed should be established stepwise; otherwise, it could induce severe adverse effects in fish and cause a big economic loss. Herein, we chose a 30% level of FM replacement to make a new feed formulation at the initial stage.

Gut microbiota are critical to the host's nutrition, development, immunity, and resistance against stressful conditions (Wang, Ran, Ringø, & Zhou, 2018). The advent of next‐generation sequencing (NGS) enabled more sophisticated analysis of complex gut microbiota by a culture‐independent approach with unprecedented resolution and throughput (Jovel et al., 2016). The NGS technique has been used to explore the dietary effects on gut microbiota of different fish species, including rainbow trout (*Oncorhynchus mykiss*; Desai et al., 2012), sea bream (*Sparus aurata*; Estruch et al., 2015), Arctic charr (*Salvelinus alpinus*; Nyman, Huyben, Lundh, & Dicksved, 2017), field eel (*Monopterus albus*; Peng et al., 2019), yellowtail kingfish (*Serio lalalandi*; Soriano et al., 2018), and channel catfish (*Ictalurus punctatus*; Wang et al., 2019). Most of these studies investigated the dietary effects on fish gut microbiota for a short‐term administration, but the long‐term dietary effects at different growth stages have generally been overlooked. Only recently, Ceppa et al. (2018) investigated the concomitant effect of diet and life stages on modulation of the gut microbiota in rainbow trout (*O. mykiss*). They identified significant differences in gut microbial composition between juvenile and adult fish supplemented with essential oil. Hitherto the dietary effect of plant protein ingredients on gut microbiota of the olive flounder has not been investigated.

The introduction of new FM alternatives in the fish diet needs to be carefully assessed at different growth stages, since diet and age are very important factors putting selective pressure on the gut microbial composition in fish (Egerton, Culloty, Whooley, Stanton, & Ross, 2018). Hence, we investigated the gut microbiota of olive flounder at different growth stages with long‐term dietary administration of plant‐based low FM and practical FM diets by a culture‐independent metagenomic approach.

## 2 MATERIALS AND METHODS

### 2.1 Experimental diets, fish, and conditions

In the experiment, we formulated two isonitrogenous and isolipidic diets as a fish meal (FM)‐based control diet (Con) and a plant‐protein‐based low FM diet (FM30) with 30% FM replacement using soybean meal, corn gluten meal, and corn concentrate (Lee et al., 2019). The ingredients and nutrient composition are shown in Table 1. The two diets were produced by thoroughly mixing the feed ingredients, following the extrusion process in a twin‐screw extruder (ATX‐II; Fesco Precision Co.) in the following conditions: feeder supply speed, 70 kg/h; conditioner temperature, 80°C; barrel temperature, 120–130°C; and main screw speed, 650 rpm. The pellets were then air‐dried at 60°C for 3 hr and stored at −20°C until use.

**Table 1:** Ingredients and nutrient composition of the experimental diets

	Control diet	FM30 diet
Ingredients (%, DM)		
Fish meal^a^	74.4	52.1
Defatted soybean meal	—	6.6
Corn gluten meal	—	6.6
Corn concentrate	—	8.8
Krill	2.0	2.0
Wheat flour	19.0	18.5
Fish oil	2.4	3.1
Vitamin E	0.2	0.2
Vitamin C	0.3	0.3
Vitamin premix^b^	0.5	0.5
Mineral premix^c^	0.5	0.5
Choline chloride	0.2	0.2
Monocalcium phosphate	0.5	0.5
Taurine	—	0.1
Nutrients (%, DM)		
Moisture	3.11	2.88
Crude protein	55.10	55.54
Crude lipid	8.66	7.53
Ash	13.15	9.43

Abbreviation: FM30, fish meal substituted with plant‐based protein sources including defatted soybean meal, corn gluten meal, and corn concentrate up to 30%.

a North Chilean Fish meal; Cia. Pesquera Camanchaca S.A.

b Vitamin premix (as g/kg premix): l‐ascorbic acid, 121.2; DL‐α‐tocopheryl acetate, 18.8; thiamin hydrochloride, 2.7; riboflavin, 9.1; pyridoxine hydrochloride, 1.8; niacin, 36.4; Ca‐d‐pantothenate, 12.7; myo‐inositol, 181.8; d‐biotin, 0.27; folic acid, 0.68; p‐aminobenzoic acid, 18.2; menadione, 1.8; retinyl acetate, 0.73; cholecalciferol, 0.003.

c Mineral premix (as g/kg premix): NaCl, 43.3; MgSO_4_·7H_2_O, 136.5; NaH_2_PO_4_·2H_2_O, 86.9; KH_2_PO_4_, 239; CaHPO_4_, 135.3; Ferric citrate, 29.6; ZnSO_4_·7H_2_O, 21.9; Ca‐lactate, 304; CuCl, 0.2; AlCl_3_·6H_2_O, 0.15; KI, 0.15; MnSO_4_·H_2_O, 2.0; CoCl_2_·6H_2_O, 1.0.

**Figure 1:**
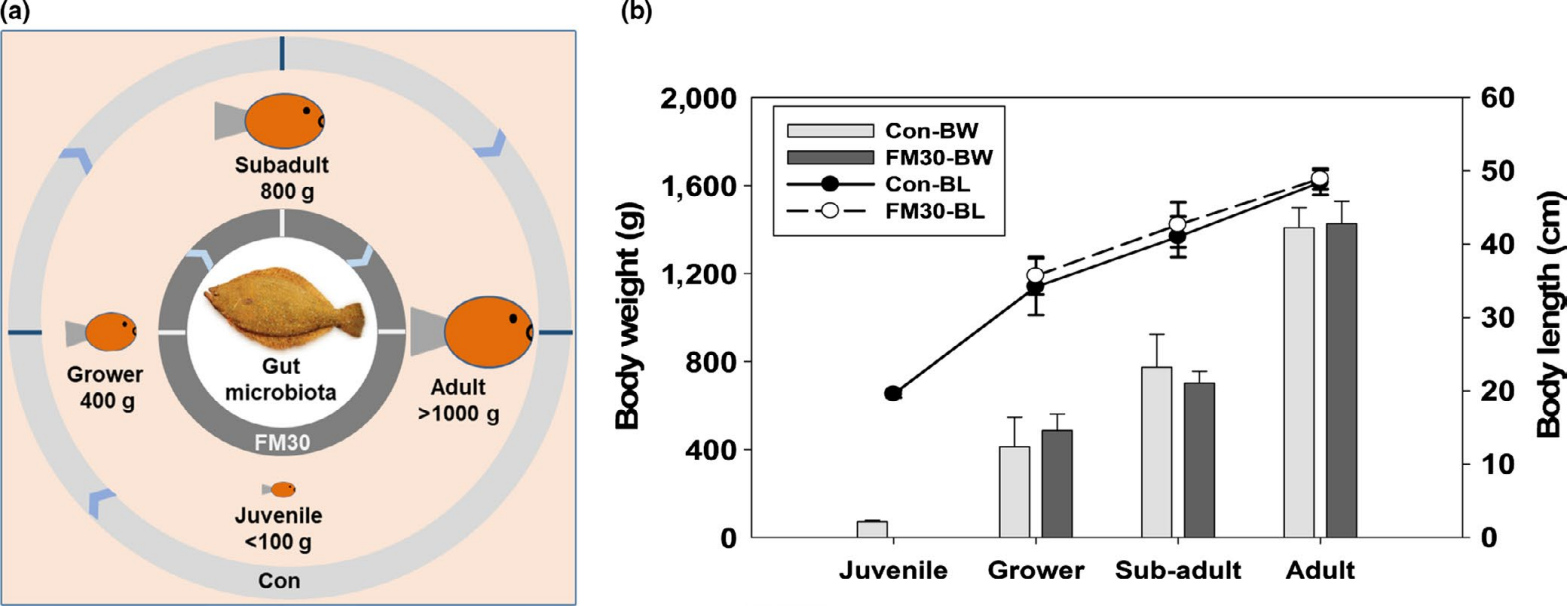
Growth stage and diet‐based gut bacterial community in olive flounder. (a) Schematic representation for the overall study; Con, a fish meal‐based control diet; FM30, a low fish meal diet containing a blend of plant proteins replacing 30% of fish meal (FM30); (b) average body weight (BW) and length (BL) of the used fish samples

The feeding trial was conducted at Aquafeed Research Center (Pohang), National Institute of Fisheries Science (NIFS), Republic of Korea, following the regulations of the Care and Use of Laboratory Animals of the NIFS with approval number as 2016‐NIFS‐IACUC‐06. We obtained juvenile olive flounder (average initial body weight, 30 g) from Korea NIFS and acclimatized to environmental conditions for 8 weeks supplied with the Con diet prior to the experiment. After the acclimatization, a total of 300 fish in each treatment fed with the Con or FM30 diet were randomly distributed into three polyvinyl circular tanks (100 fish/tank; volume, 400 L) supplied with seawater at a flow rate of 20 L/min and aeration. The fish were reared in an indoor flow‐through system with standard conditions, and the water temperature ranged from 16.8 to 26.1°C. The feeding trial was conducted for 8 months, and all the fish were fed twice a day at the ad libitum level. Three fish with a body weight of <100 g were collected before the initiation of the experiment and were assigned as the juvenile stage (<100 g). After the start of the experiment, we collected two fish per tank after 2, 4, and 8 months of rearing and measured them for body weight (BW) and length (BL). On the basis of the Standard Manual of Olive Flounder Culture (National Fisheries Research and Development Institute (NFRDI), 2006) and Okorie et al. (2014), we designated the collected fish as the grower (~400 g), subadult (~800 g), and adult (>1,000 g) growth stage based on their BW and BL, as shown in Figure 1.

### 2.2 Sample collection, DNA extraction, and sequencing

The midgut contents of the olive flounders (*P. olivaceus*) were collected using the method described by Kim, Brunt, and Austin (2007). Briefly, we used a scalpel to separate the digestive tract from the abdominal cavity aseptically, following the contents of midguts were squeezed and stored in microtubes. The extraction and purification of the genomic DNA in the fish gut content as well as the amplification of V3–V4 hypervariable region of the bacterial 16S rRNA gene were conducted as described previously (Niu et al., 2019).

### 2.3 Sequencing data analysis

The MiSeq raw data were changed, processed, and qualified to obtain high‐quality sequences by removing the sequencing errors, ambiguous sequences, and chimerical sequences using a series of programs as described previously (Li & Durbin, 2009; Magoč & Salzberg, 2011; Zhang, Schwartz, Wagner, & Miller, 2000). The diversity analyses based on the operational taxonomic unit (OTU) data were performed using QIIME (v1.8; Caporaso et al., 2010) and according to the previous method (Niu et al., 2019).

### 2.4 Statistical analysis

Canonical correspondence analysis was used to conduct the statistically significant differences in the environmental parameters of samples at *p* < .05 in R (v 3.1.2). The α‐ and β‐diversities with the phylum level of microbiota based on growth stage and diet type were analyzed by one‐way ANOVA (Analysis of variance) in SPSS version 24 (SPSS IBM, New York, USA) with *p* < .05. In addition, α‐diversity with genus level between the Con and FM30 diet groups was assessed by the Mann–Whitney *U* test in SPSS.

## 3 RESULTS

### 3.1 Basic features and diversity analysis

**Figure 2:**
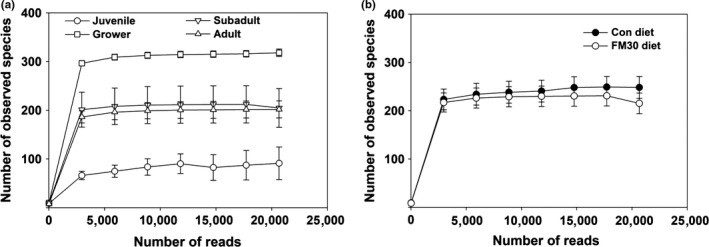
Rarefaction analysis on the gut bacterial community of olive flounder with respect to (a) growth stage and (b) diet type

**Figure 3:**
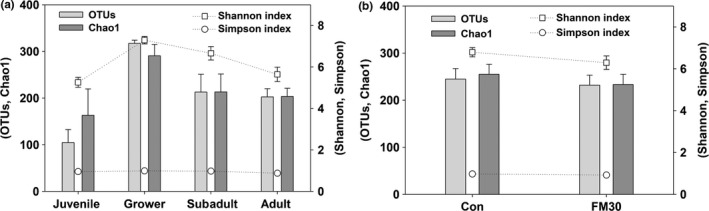
Alpha diversity analysis on the gut bacterial community of olive flounder with respect to (a) growth stage and (b) diet type

**Figure 4:**
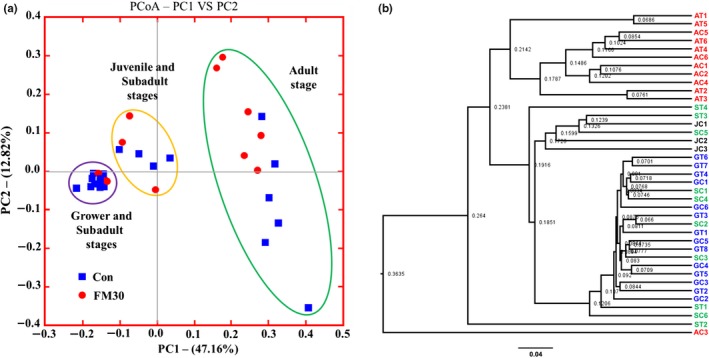
Beta diversity analysis on the gut bacterial community of olive flounder. (a) PCoA plot and (b) UPGMA phylogenetic tree. AC, adult fish fed with the control diet; AT, adult fish fed with the treatment diet (FM30); GC, grower fish fed with the control diet; GT, grower fish fed with the treatment diet (FM30); JC, juvenile fish fed with the control diet; SC, subadult fish fed with the control diet; ST, subadult fish fed with the treatment diet (FM30); Unknown, sequences that could not be classified into any known group were labeled as “Unknown”

A total of 4,695,029 reads were identified from the gut samples of a total of 39 olive flounder (*P. olivaceus*) fed with the Con and FM30 diets. After trimming, processing, and removing chimera sequences, we obtained a total of 1,128,916 valid reads with 29,517 median reads in all the samples. The observed species following the sequencing reads increased and kept constant after 5,000 reads. During growth, the fish in the grower stage showed the highest number of species, followed by subadult, adult, and juvenile stages (Figure 2a). A similar number of species was observed in the gut microbiota of fish fed with the Con and FM30 diets (Figure 2b). The remaining filtered sequences were further used to analyze the alpha and beta diversities. Apparently, the species richness based on the OTUs and Chao 1 indexes presented an increased trend from the juvenile stage to the grower stage, followed by a decrease in the subadult and adult stages. A similar change was also observed in the Shannon index of the gut microbial diversity, whereas the Simpson index showed no change during the growth (Figure 3a). The α‐diversity based on the diet factor showed similar values involving the OTUs, Chao1, Shannon, and Simpson index (Figure 3b). The diet factor affected the α‐diversity of the gut microbiota of olive flounder less than did the growth factor. The β‐diversity of the fish gut microbiota was displayed by a PCoA plot and UPGMA tree (Figure 4). The gut microbial communities were clustered into three main groups based on the growth stage, but the difference among them was presented only at the adult stage fed with Con and FM30 diets.

### 3.2 The dominant gut microbiota

**Figure 5:**
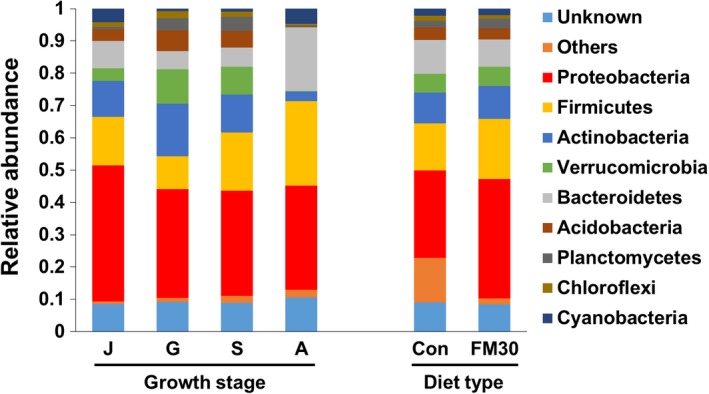
Relative abundance of bacterial phyla in different growth stages of olive flounder fed with control and low FM (FM30) diets. A, adults; G, growers; J, juveniles; S, subadults

At the phylum level, there were nine dominant phyla found in the gut microbiota of olive flounder including Proteobacteria, Firmicutes, Actinobacteria, Bacteroidetes, Cyanobacteria, Verrucomicrobia, Acidobacteria, Chloroflexi, and Planctomycetes. Of these phyla, Proteobacteria was the most abundant, regardless of the difference in diet and growth stage (Figure 5). During growth, the abundance of Firmicutes and Bacteroidetes displayed an increased trend; in contrast, the abundance of Actinobacteria and Verrucomicrobia showed a decreased trend. In terms of diet, there was no significant difference on altering the relative abundance of the nine dominant phyla in the gut bacterial community of olive flounder. At the genus level (≥1% relative abundance), the five most dominant genera in juvenile fish were *Deltia* (12.62 ± 3.8%), *Prevotella* (3.98 ± 1.73%), *Chthoniobacter* (3.65 ± 0.89%), *Acetobacter* (3.14 ± 2.01%), and *Lactobacillus* (3.08 ± 1.63%); in grower fish were *Chthoniobacter* (8.17 ± 0.44%), *Bacillus* (5.57 ± 0.40%), *Bradyrhizobium* (3.41 ± 0.22%), *Rhodoplanes* (2.80 ± 0.08%), and one unclassified (2.61 ± 0.20%); in subadult fish were *Chthoniobacter* (6.92 ± 1.28%), *Bacillus* (5.28 ± 0.68%), *Delftia* (5.21 ± 1.19%), *Lactobacillus* (4.86 ± 1.19%), and *Bradyrhizobium* (2.55 ± 0.68%); in adult fish were *Lactobacillus* (10.35 ± 4.48%), *Prevotella* (9.48 ± 1.40%), one unclassified (3.91 ± 0.35%), *Paraprevotella* (1.22 ± 0.48%), and *Bacillus* (1.14 ± 0.24%; Table 2). The gut microbial diversity in the adult growth stage presented less number of genera (>1% relative abundance) compared with that of other growth stages. In comparison with the gut bacterial community of fish fed with the Con and FM30 diets, the significant difference was mainly observed in the abundance of *Prevotella*, *Photobacterium*, *Lactobacillus*, *Paraprevotella*, *Capnocytophaga*, *Propionibacterium*, and *Rhodopila*. The six most dominant genera were *Chthoniobacter* (5.42 ± 0.93%), *Prevotella* (4.77 ± 1.40%), *Bacillus* (4.18 ± 0.59%), *Delftia* (3.44 ± 1.05%), *Photobacterium* (3.02 ± 1.99%), and *Lactobacillus* (2.51 ± 0.98%) in fish fed with the Con diet and *Photobacterium* (10.90 ± 4.96%), *Lactobacillus* (7.40 ± 3.20%), *Chthoniobacter* (4.49 ± 0.99%), *Bacillus* (3.54 ± 0.56%), *Prevotella* (3.12 ± 0.80%), *Delftia* (2.71 ± 0.78%) in fish fed with the FM30 diet (Table 3).

**Table 2:** A growth‐related taxonomic profile (genus level, ≥1% relative abundance) of the gut bacterial community in olive flounder

Growth stage											
Juvenile			Grower			Subadult			Adult		
Genus (phylum)	Ave	*SEM*	Genus (phylum)	Ave	*SEM*	Genus (phylum)	Ave	*SEM*	Genus (phylum)	Ave	*SEM*
*Delftia* (Pro)	12.62	3.80	*Chthoniobacter* (Ver)	8.17	0.44	*Chthoniobacter* (Ver)	6.92	1.28	*Lactobacillus* (Fir)	10.35	4.48
*Prevotella* (Bac)	3.98	1.73	*Bacillus* (Fir)	5.57	0.40	*Bacillus* (Fir)	5.28	0.68	*Prevotella* (Bac)	9.48	1.40
*Chthoniobacter* (Ver)	3.65	0.89	*Bradyrhizobium* (Pro)	3.41	0.22	*Delftia* (Pro)	5.21	1.19	Unclassified (Cya)	3.91	0.35
*Acetobacter* (Pro)	3.14	2.01	*Rhodoplanes* (Pro)	2.80	0.08	*Lactobacillus* (Fir)	4.86	2.16	*Paraprevotella* (Bac)	1.22	0.48
*Lactobacillus* (Fir)	3.08	1.63	Unclassified (Act)	2.61	0.20	*Bradyrhizobium* (Pro)	2.55	0.68	*Bacillus* (Fir)	1.14	0.24
Unclassified (Act)	2.65	1.62	*Actinoallomurus* (Act)	2.52	0.16	*Rhodoplanes* (Pro)	2.41	0.35	*Staphylococcus* (Fir)	1.07	0.36
*Bacillus* (Fir)	2.37	1.34	*Pseudomonas* (Pro)	2.51	0.24	*Candidatus Solibacter* (Aci)	2.21	0.35	*Pseudomonas* (Pro)	1.07	0.23
*Escherichia* (Pro)	2.28	0.69	*Candidatus Solibacter* (Aci)	2.47	0.17	*Actinoallomurus* (Act)	2.11	0.20			
*Serratia* (Pro)	2.06	1.05	*Delftia* (Pro)	2.03	0.20	Unclassified (Act)	1.70	0.48			
*Actinomadura* (Act)	1.90	0.02	*Actinomadura* (Act)	1.95	0.15	*Prevotella* (Bac)	1.67	0.85			
*Paraprevotella* (Bac)	1.88	0.94	*Candidatus Koribacter* (Aci)	1.81	0.17	*Pseudomonas* (Pro)	1.64	0.32			
*Candidatus Koribacter* (Aci)	1.61	0.94	Unclassified (Pro)	1.66	0.21	*Staphylococcus* (Fir)	1.57	1.06			
Unclassified (Pro)	1.57	0.84	*Conexibacter* (Act)	1.42	0.15	Unclassified (Pla)	1.46	0.46			
Unclassified (Pro)	1.52	1.00	*Gemmata* (Pla)	1.26	0.16	Clostridium (Fir)	1.38	0.44			
*Bradyrhizobium* (Pro)	1.46	0.84	Unclassified (Pro)	1.11	0.12	*Candidatus Koribacter* (Aci)	1.25	0.32			
*Sphingomonas* (Pro)	1.24	1.05	*Solirubrobacter* (Act)	1.06	0.15	*Acinetobacter* (Pro)	1.15	0.63			
Unclassified (Pro)	1.17	0.69	Unclassified (Pro)	1.04	0.19	*Acetobacter* (Pro)	1.13	0.39			
*Propionibacterium* (Act)	1.17	0.16				*Conexibacter* (Act)	1.11	0.21			
*Lysinibacillus* (Fir)	1.16	1.16				*Actinomadura* (Act)	1.08	0.32			
*Staphylococcus* (Fir)	1.04	0.63				Unclassified (Pro)	1.00	0.12			

Abbreviations: Aci, Acidobacteria; Act, Actinobacteria; Bac, Bacteroidetes; Cya, Cyanobacteria; Fir, Firmicutes; Pla, Planctomycetes; Pro, Proteobacteria; Ver, Verrucomicrobia.

**Table 3:** A diet‐related taxonomic profile (genus level, ≥1% relative abundance) of the gut bacterial community in olive flounder

Genus (phylum)	Con		FM30		*p* Value
	Ave.	*SEM*	Ave.	*SEM*	
*Chthoniobacter* (Ver)	5.42	0.93	4.49	0.99	.670
*Prevotella* (Bac)	4.77	1.26	3.12	0.80	.022
*Bacillus* (Fir)	4.18	0.59	3.54	0.56	.651
*Delftia* (Pro)	3.44	1.05	2.71	0.78	.341
*Photobacterium* (Pro)	3.02	1.99	10.90	4.96	.002
*Lactobacillus* (Fir)	2.51	0.98	7.40	3.20	.015
*Bradyrhizobium* (Pro)	1.99	0.40	1.99	0.46	.435
Unclassified (Cya)	1.88	0.60	1.47	0.37	.048
Unclassified (Act)	1.86	0.37	1.26	0.32	.294
*Pseudomonas* (Pro)	1.75	0.27	1.54	0.24	.094
*Candidatus Solibacter* (Aci)	1.70	0.31	1.30	0.26	.115
*Rhodoplanes* (Pro)	1.64	0.32	1.70	0.32	.556
*Actinoallomurus* (Act)	1.47	0.26	1.61	0.29	.779
*Actinomadura* (Act)	1.22	0.21	1.01	0.26	.448
*Candidatus Koribacter* (Aci)	1.18	0.24	0.99	0.23	.700
Unclassified (Pro)	1.07	0.21	0.90	0.18	.360
*Staphylococcus* (Fir)	0.96	0.23	0.87	0.60	.506
*Paraprevotella* (Bac)	0.94	0.32	0.14	0.08	.000
Unclassified (Pro)	0.85	0.18	0.55	0.14	.134
Unclassified (Bac)	0.83	0.24	0.54	0.15	.049
*Gemmata* (Pla)	0.82	0.18	0.55	0.14	.174
*Acetobacter* (Pro)	0.81	0.34	0.53	0.27	.642
*Bacteroides* (Bac)	0.74	0.25	0.76	0.24	.908
*Conexibacter* (Act)	0.73	0.15	0.94	0.21	.167
*Clostridium* (Fir)	0.70	0.21	0.55	0.19	.645
Unclassified (Pro)	0.65	0.17	0.54	0.18	.780
Unclassified (Pla)	0.59	0.13	0.97	0.31	.006
*Sphingomonas* (Pro)	0.59	0.15	0.50	0.10	.831
*Capnocytophaga* (Bac)	0.58	0.29	0.18	0.07	.022
Unclassified (Fir)	0.57	0.17	0.41	0.13	.265
*Escherichia* (Pro)	0.56	0.19	0.27	0.13	.142
Unclassified (Act)	0.55	0.12	0.30	0.10	.216
Unclassified (Fir)	0.55	0.18	0.26	0.08	.004
*Propionibacterium* (Act)	0.55	0.10	0.31	0.07	.028
Unclassified (Pro)	0.55	0.10	0.60	0.13	.410
Unclassified (Pro)	0.54	0.24	0.43	0.15	.536
*Rhodopila* (Pro)	0.53	0.15	0.20	0.06	.008
*Stella* (Pro)	0.51	0.12	0.46	0.13	.938

Abbreviations: Aci, Acidobacteria; Act, Actinobacteria; Bac, Bacteroidetes; Cya, Cyanobacteria; Fir, Firmicutes; Pla, Planctomycetes; Pro, Proteobacteria; Ver, Verrucomicrobia.

## 4 DISCUSSION

Gut microbiota plays important roles in nutritional, functional, and physiological activities of the host. Several factors including intrinsic (i.e., age) and extrinsic (i.e., diet) may affect the fish gut microbial diversity, function, and metabolic activities. To date, little information regarding the change in gut microbiota at different growth stages of olive flounder was reported. Therefore, understanding its composition in response to diet change over its lifetime will be very valuable for establishing practical low FM aquafeeds for olive flounder. According to our findings, the gut bacterial composition was not significantly influenced by diet until the adult stage, whereas obvious shifts of the gut bacterial community were observed at different growth stage. The microbiota of the grower fish was characterized by the highest α‐diversity measurements, such as number of observed species and the OTUs, Chao1, and Shannon indexes, in contrast to juvenile, subadult, and adult fish. Generally, a high diversity is regarded beneficial for host health (Fan et al., 2019). A recent study by Ceppa et al. (2018) in rainbow trout also observed less species richness in juvenile than in the adult fish. However, it is contradicted by the study of Stephens et al. (2016), which reported the decreased OTU richness of the gut microbiota throughout the development of zebra fish. Measures of β‐diversity can elucidate how much diversity is unique to a local assemblage or to ecological processes, such as habitat filtering or competition (Lozupone & Knight, 2008). We observed that the bacterial communities were distinctly grouped according to their growth stages, but were in close relationship among the individuals of different dietary groups. Overall, in comparison with the dietary effect, there was clear modulation of the growth stage on the gut microbiota in olive flounder. Ceppa et al. (2018) and Fan et al. (2019) also observed no significant effects of the dietary treatments on the gut microbiota in rainbow trout and shrimp, respectively; however, they observed significant differences in the gut bacterial community at different growth stages. Another study reported the replacement of FM with the plant proteins (at 30% inclusion) had no significant effect on the levels of total aerobic and anaerobic bacterial counts in the intestine of silver crucian carp (*Carassius auratus gibelio* × *Cyprinus carpio*; Cai et al., 2012).

Regardless of growth stage and diet, the gut microbiota in olive flounder are mainly dominated by four phyla, namely Proteobacteria, Firmicutes, Actinobacteria, and Bacteroidetes which is in general agreement with the previous results based on a culture‐dependent method in wild and farmed olive flounder (~300 g; grower; Kim & Kim, 2013). In addition, some other subdominant phyla, namely Verrucomicrobia and Acidobacteria, were also found in our samples. Notably, the composition of gut microbiota in the same fish species might be sometimes difficult to compare between the studies, since many factors, such as DNA extraction methods or time of DNA extraction, can also influence these communities. Herein, from grower to adult fish, the phylum Firmicutes showed a continuous increase in relative abundance, with *Bacillus* as the major representative genus. Desai et al. (2012) also observed that 30% SBM inclusion in the diets of rainbow trout led to an increase in Firmicutes. *Bacillus* species are gram‐positive, spore‐forming bacteria; many strains are typically used as commercial probiotics (Wang, Li, & Lin, 2008). Previously, Aly, Ahmed, Ghareeb, and Mohamed (2008) also suggested *Bacillus subtilis* as a potential probiotic for the growth in *Oreochromis niloticus* because of its antimicrobial activity against bacterial pathogens. Cha, Rahimnejad, Yang, Kim, and Lee (2013) evaluated the dietary supplementation of *Bacillus* strains in olive flounder and its response to infection with *Streptococcus iniae*. Fish fed *Bacillus* showed significantly higher survival rates; however, the underpinning mechanism remains elusive. For the effect of a low FM diet, it was observed that *Photobacterium* (Proteobacteria) and *Lactobacillus* (Firmicutes) were also sharply increased at the adult stage, especially in the FM30 group. The *Photobacterium* mainly consist of *Photobacterium piscicola*, which are commonly found on the surface or the intestine of healthy fish as mutualistic bacteria and could produce diverse enzymes (Figge et al., 2014). The higher abundance of *Lactobacillus* in adult olive flounder fed on low FM diet has also been reported in salmonids and gilthead sea bream previously (Desai et al., 2012; Gajardo et al., 2017; Parma et al., 2016; Reveco, Øverland, Romarheim, & Mydland, 2014). Theilmann et al. (2017) suggested that *Lacotobacillus* can utilize plant glycosides (PGs) because of the conserved PG‐usage gene loci of the phosphotransferase systems (PTS) transporters and phospho‐β‐glucosidases. Thus, in the context of our research, we hypothesize that the *Lactobacillus* growth could be supported by these plant glycosides. The functional impact of *Lactobacillus* on fish intestine is still unclear, but potentially they may have beneficial effects on the immune system, protect fish from pathogens, and are common probiotic candidates (Parma et al., 2016). In this study, Firmicutes and Proteobacteria were identified as biomarkers in different developmental stages in relation to low FM diet, suggesting that these phyla were prevalent in the gut microbiota of olive flounder and different species of these phyla may perform different functions in the gut ecosystem. However, further studies are warranted on the functions of these gut microbes to understand their roles in the gut of olive flounder.

## 5 CONCLUSIONS

Our study displays the gut microbial profile of farmed olive flounder with long‐term administration of a low FM diet using an NGS method for the first time. We have found a close relationship between the gut microbial composition and growth stage of olive flounder. The FM30 diet had subtle effects on altering the gut microbiota in the early growth stage of olive flounder. However, the abundance of *Lactobacillus* and *Photobacterium* was significantly increased after the FM30 administration for 8 months. These results could possibly provide valuable information to establish a successful low or free‐FM aquafeed for the host. Further studies need to delineate the specific changes in the overall health of the host, including growth performance, immune response, mortality, physiological parameters, and functional genomics, in response to the low FM diet across different growth stages.

## CONFLICT OF INTEREST

None declared.

## AUTHOR CONTRIBUTIONS

Kai Min Niu: Conceptualization‐Equal, Data curation‐Lead, Formal analysis‐Lead, Investigation‐Equal, Methodology‐Lead, Software‐Lead, Writing‐original draft‐Lead, Writing‐review & editing‐Equal; Bong‐Joo Lee: Conceptualization‐Equal, Funding acquisition‐Lead, Methodology‐Equal, Project administration‐Equal, Resources‐Equal, Writing‐review & editing‐Equal; Damini Kothari: Data curation‐Equal, Formal analysis‐Equal, Writing‐original draft‐Equal, Writing‐review & editing‐Equal; Woo‐Do Lee: Data curation‐Supporting, Investigation‐Supporting, Methodology‐Supporting, Resources‐Supporting; Sang‐Woo Hur: Funding acquisition‐Equal, Resources‐Supporting; Sang‐Gu Lim: Funding acquisition‐Supporting, Resources‐Supporting, Supervision‐Equal; Kang Woong Kim: Funding acquisition‐Equal, Resources‐Supporting; Kyoung‐Duck Kim: Investigation‐Equal, Resources‐Equal; Na‐Na Kim: Conceptualization‐Supporting, Investigation‐Equal, Writing‐review & editing‐Supporting; Soo‐Ki Kim: Conceptualization‐Lead, Project administration‐Lead, Supervision‐Lead.

## ETHICS STATEMENT

The intestinal contents of fishes were collected by Aquafeed Research Center, National Institute of Fisheries Science (Pohang, Republic of Korea), following the guidelines of the Animal Ethics Committee Regulations (2016‐NIFS‐IACUC‐06).

## Data Availability

The datasets generated and analyzed during the current study are available in the figshare repository at https://doi.org/10.6084/m9.figshare.10007462.
